# Data sets on human histone interaction networks

**DOI:** 10.1016/j.dib.2020.106555

**Published:** 2020-11-21

**Authors:** Yunhui Peng, Yaroslav Markov, Alexander Goncearenco, David Landsman, Anna R. Panchenko

**Affiliations:** a National Center for Biotechnology Information, National Institutes of Health, Bethesda, MD, United States; b Computational Biology and Bioinformatics, Combined Program in the Biological and Biomedical Sciences, Yale University, New Haven, CT, United States; c VantAI, New York, NY, United States; d Department of Pathology and Molecular Medicine, School of Medicine, Queen's University, ON, Canada

**Keywords:** Histone interaction, Nucleosome interaction, Interactome, Interaction network, Histone variant

## Abstract

Here, we present the data of human histone interactomes generated and analysed in the research article by Peng et al., 2020 [1]. The histone interactome data provide a comprehensive mapping of human histone/nucleosome interaction networks by using different data sources from the structural, chemical cross-linking, and high-throughput studies. The histone interactions are presented at different levels of granularity in networks, including protein, domain, and residue-levels. All human histone interactome Cytoscape session files are available at https://github.com/Panchenko-Lab/Human-histone-interactome.

## Specifications Table

**Table:** 

Subject	Chromatin Biology
Specific subject area	Interaction network of human histones
Type of data	Table, Cytoscape session files
How data were acquired	PDB structures(https://www.rcsb.org), APID datasets (http://apid.dep.usal.es), Cross-linking mass spectrometry data (https://doi.org/10.1074/mcp.RA118.000924)
Data format	Raw and Analysed
Parameters for data collection	Histone interaction interface from PDB structures: at least one heavy atom of histone residue is within 5 Å of any heavy atoms of a binding partner. Histone interaction from cross-linking data: human histone is crosslinked with another non-histone human protein. Histone interaction from high-throughput data: human histone interaction supported by “binary” methods in APID database.
Description of data collection	Human histone interactions in structural interactome are collected from the available histone and nucleosome complex structures in PDB [2]. Human histone cross-linking interactomes are constructed using the histone interactions observed from the human nuclei cross-linking study [3]. Human histone high-throughput interactomes are constructed by extracting human histone interactions from the APID database supported by “binary” methods that provide data on direct physical interactions between proteins [4].
Data source location	Online at Github: https://github.com/Panchenko-Lab/Human-histone-interactome Primary data sources: PDB structures: https://www.rcsb.org APID datasets: http://apid.dep.usal.es Cross-linking mass spectrometry data: https://doi.org/10.1074/mcp.RA118.000924
Data accessibility	https://github.com/Panchenko-Lab/Human-histone-interactome
Related research article	Peng Y, Markov Y, Goncearenco A, Landsman D, Panchenko AR. Human histone interaction networks: an old concept, new trends. J Mol Biol. In Press. https://doi.org/10.1016/j.jmb.2020.10.018

## Value of the Data

• These data provide a comprehensive mapping of human histone interaction networks.
• Datasets on human histone interactomes allow to characterize the properties of histone interactions and elucidate the mechanisms of regulatory processes associated with histones.
• Human structural and cross-linking interactomes identify binding hotspots and variant-specific interactions among all histone families.
• These data provide the classification of functions of various histone binding proteins.

## 1 Data Description

We present the histone interaction networks which are constructed and analyzed in detail in Ref. [1]. Table 1 gives the list of constructed human histone interactomes. Table 2 summarizes the information on all histone global interactomes, which are defined by combining the histone interaction network with an additional layer of partners interacting with histone-binding proteins. All constructed interactomes are saved as Cytoscape session files and archived at https://github.com/Panchenko-Lab/Human-histone-interactome, which can be read with Cytoscape 3.7.0 or later versions [5].

**Table 1:** Data files for human histone interactomes at different levels of granularity (protein-level, domain-level and residue-level).

File name	Description
Structural_protein.cys	Protein-level structural interactome
Structural_nucleosome_protein.cys	Protein-level structural interactome that only contains interactions extracted from nucleosome complex structures
Structural_domain.cys	Domain-level structural interactome
Structural_residue.cys	Residue-level structural interactome
Crosslinking_protein.cys	Protein-level cross-linking interactome
Crosslinking_domain.cys	Domain-level cross-linking interactome
Crosslinking_residue.cys	Residue-level cross-linking interactome
High-throughput_protein.cys	Protein-level high-throughput interactome

**Table 2:** Data files for human histone global interactomes at protein-level.

File name	Description
Structural_global_protein.cys	Protein-level structural global interactome
Crosslinking_global_protein.cys	Protein-level cross-linking global interactome
High-throughput_global_protein.cys	Protein-level high-throughput global interactome
combine_structural_crosslinking.cys	Protein-level global interactome combined from structural and cross-linking data
combine_all.cys	Protein-level global interactome combined from structural, cross-linking, and high-throughput data

## 2 Experimental Design, Materials and Methods

### 2.1 Histone structural interactome

The human histone structural interactome comprises histone interactions extracted from all available histone and nucleosome complex structures in PDB [2]. The following protocols described how we built the histone structural interactome: first, we performed the text search against PDB with a list of histone-associated keywords including “Histone”, “H1”, “H2A”, “H2B”, “H3”, “H4”, “H5”, “CENP-A”. We further used the PDB identifiers of obtained structures to extract the information about species, protein names, UniProt accessions, and chain identifiers using the RCSB PDB RESTful Web Service interface (https://www.rcsb.org/pdb/software/rest.do). Finally, we applied three stages of filtering to extract the complex structures of human histones or nucleosomes: i) structures without human histones were excluded; ii) we removed the ambiguous cases of synthetic constructs that do not have relevant UniProtKB accessions and could not be mapped to histone sequences in HistoneDB 2.0 database [6, 7]; iii) structures without protein binding partners were excluded. We kept the inter-species histone interactions if their binding partners were evolutionary conserved. Unique histone-binding partners were defined as proteins with different UniProtKB accession numbers. In total, there are 208 histone interactions with 164 different binding proteins from 345 structures of histone or nucleosome complexes (Table 1).

To identify binding interfaces, coordinates of biological assemblies were retrieved from the PDB in PDBx/mmCIF format for analysis of inter-protein contacts. Interfaces were defined as residues located within 5 Å distance between heavy atoms of histones and their binding partners. Then we mapped residue locations to the sequences of the corresponding UniProtKB entries by SIFTS [8]. Next, we identified domain families of histone-binding proteins using protein domain family annotations from the Conserved Domain Database, which represents a collection of manually annotated multiple sequence alignment models for protein domain families and full-length proteins [9]. Proteins from the same domain family were grouped together in the “domain-level network”, which includes 137 interactions from 113 unique conserved domain families.

### 2.2 Histone cross-linking interactome

We extracted histone interactions between human histones crosslinked with another human non-histone protein using interaction data set from both fractionated and unfractionated cross-linked nuclei [3]. It includes totally 1855 protein-protein interactions (PPIs) in nuclei. There are 274 interactions from 200 different histone-binding proteins in this so-called “cross-linking histone network”. Domain annotations from the Conserved Domain Database [9] were further used to construct the domain-level cross-linking network, including 107 interactions from 70 different domain families. Finally, we extracted binding interfaces by mapping lysine residues forming inter-protein cross-links onto protein sequences to build the residue-level network [3].

### 2.3 Histone high-throughput interactome

APID database comprises protein-protein interactions from several major databases of molecular interactions from more than 1100 organisms [4]. We extracted human histone interactions from the APID database, which were supported by direct physical interactions verified by “binary” methods. Then, inter-species interactions were not included in constructed networks. Human histones were identified using UniProtKB accessions and histone interactions were identified between human histones interacting with human non-histone proteins. As a result, this data set includes 220 interactions between histones and 163 non-histone proteins. APID database did not give sufficient information to build the domain- and residue- levels histone interaction networks since no data on binding interfaces were available for most entries.

### 2.4 Histone global interactome

We further included an additional layer of those proteins that interacted with histone-binding proteins in PDB structures, cross-linking mass spectrometry data, and APID database. This is so-called “histone global interactome” (Table 2). We further combined the global networks of structural and cross-linking interactomes, which comprised 987 edges and 754 nodes from 353 domain families (nodes with the same UniProtKB accessions were grouped together). Finally, we integrated interactions from all structural, cross-linking, and high-throughput data totalling 5308 nodes and 10,330 edges.

## Ethics statement

**If the work involved the use of human subjects:** NO

**If the work involved animal experiments:** NO

**If the work involves data collected from social media platforms:** NO

## CRediT author statement

YP: Conceptualization, Methodology, Software, Validation, Investigation, Writing - Original Draft, Data Curation. YM: Conceptualization, Methodology. AG: Methodology, Software. DL: Conceptualization, Review & Editing, Supervision, Funding acquisition. ARP: Conceptualization, Writing - Review & Editing, Supervision, Funding acquisition.

## Declaration of Competing Interest

The authors declare that they have no known competing financial interests or personal relationships which have or could be perceived to have influenced the work reported in this article.
